# CD8^+^ T-cell immune escape by SARS-CoV-2 variants of concern

**DOI:** 10.3389/fimmu.2022.962079

**Published:** 2022-10-27

**Authors:** Arnaud John Kombe Kombe, Fleury Augustin Nsole Biteghe, Zélia Nelly Ndoutoume, Tengchuan Jin

**Affiliations:** ^1^ Department of Obstetrics and Gynecology, The First Affiliated Hospital of University of Science and Technology of China (USTC), Division of Life Sciences and Medicine, University of Science and Technology of China, Hefei, China; ^2^ Department of Radiation Oncology, Cedars Sinai Hospital, Los Angeles, Los Angeles, CA, United States; ^3^ The Second Clinical School, Medical Imaging, Chongqing Medical University, Chongqing, China; ^4^ Laboratory of Structural Immunology, Chinese Academic of Sciences Key Laboratory of Innate Immunity and Chronic Disease, Division of Life Sciences and Medicine, University of Science and Technology of China, Hefei, China; ^5^ Chinese Academic of Sciences (CAS) Center for Excellence in Molecular Cell Science, Chinese Academy of Science, Shanghai, China

**Keywords:** SARS-CoV-2, cellular immunity, CD8 + T-cell epitope, cytotoxic T lymphocytes (CTL), variant of concern (VOC), protein mutation, HLA, immune escape

## Abstract

Despite the efficacy of antiviral drug repositioning, convalescent plasma (CP), and the currently available vaccines against severe acute respiratory syndrome coronavirus 2 (SARS-CoV-2), the worldwide coronavirus disease 2019 (COVID-19) pandemic is still challenging because of the ongoing emergence of certain new SARS-CoV-2 strains known as variants of concern (VOCs). Mutations occurring within the viral genome, characterized by these new emerging VOCs, confer on them the ability to efficiently resist and escape natural and vaccine-induced humoral and cellular immune responses. Consequently, these VOCs have enhanced infectivity, increasing their stable spread in a given population with an important fatality rate. While the humoral immune escape process is well documented, the evasion mechanisms of VOCs from cellular immunity are not well elaborated. In this review, we discussed how SARS-CoV-2 VOCs adapt inside host cells and escape anti-COVID-19 cellular immunity, focusing on the effect of specific SARS-CoV-2 mutations in hampering the activation of CD8^+^ T-cell immunity.

## Graphical Abstract

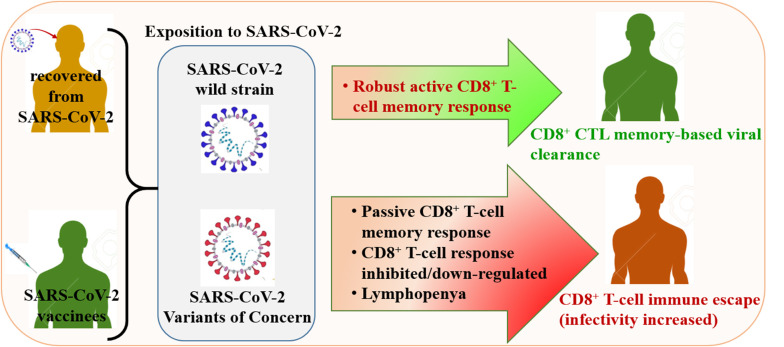

## 1 Introduction

The current pandemic of coronavirus disease 2019 (COVID-19) drives the global population in a deep phobia, as the COVID-19–associated burden is critical, resulting in thousands of deaths each day. As of 09 August 2022, there have been 590,443,154 people infected with severe acute respiratory syndrome coronavirus 2 (SARS-CoV-2), the virus responsible for COVID-19, with 6,439,059 deaths worldwide. Around 21,787,511 people are still actively infected, and approximately 0.5 million new cases and 658 new deaths are reported daily, with a re-increasing trend of new infections observed from the beginning of the 2021 winter (https://covid19.who.int/; https://www.worldometers.info/coronavirus/).

Several studies have reported that these new COVID-19 cases (or waves) are more likely to be caused by infections with emerging SARS-CoV-2 variants of concern (VOCs) (in 98% of cases) than infections with the wild-type (WT) SARS-CoV-2 strain (1–3) initially isolated in Wuhan, China in December 2019 (4, 5). Based on the Pango nomenclature system (6–9), the WHO and the CDC defined VOCs as “variants associated with a high degree of transmissibility, disease severity, neutralizing antibody and vaccine resistance, reduced treatment effectiveness, or diagnostic detection failure” (https://www.cdc.gov/coronavirus/2019-ncov/variants/variant-classifications.html#anchor_1632154493691). Indeed, people infected with emerging SARS-CoV-2 VOCs are more infectious than those infected with WT SARS-CoV-2 and other variants, suggesting that the VOCs have a higher ability to spread than the original SARS-CoV-2 strain (1, 3, 10). For instance, Daniloski et al. demonstrated that SARS-CoV-2 mutants bearing only the D614G mutation confer an increased ability to spread more quickly than the WT SARS-CoV-2 (11). Moreover, Chen et al. showed that the Omicron variant might be 10 times more infectious than the WT virus and almost three times as infectious as the Delta variant (12). Moreover, numerous reports support the fact that emerging VOCs are more severe, with a higher mortality risk than WT SARS-CoV-2, can resist prevention and treatment strategies used so far against WT SARS-CoV-2, and can escape preexisting WT SARS-CoV-2 immunity (3, 13–15). Beta, Gamma, and Omicron variants, for instance, have been shown to have reduced neutralization by monoclonal antibody therapy (including bamlanivimab and the Rockefeller University antibody C144 for Omicron), convalescent plasma (CP), and postvaccination sera (3, 16–18). Immune escape by emerging SARS-CoV-2 VOCs is, therefore, the main concern in the COVID-19 pandemic management (19).

Mutations occurring in SARS-CoV-2 spike protein may confer on VOCs the ability to adapt to and escape from natural and vaccine-induced immunity and fast spread, resulting in a detrimental effect on public health. Liu et al. (14) reported that mutations in the receptor binding domain (RBD) and N-terminal domain (NTD) play a crucial role in variant resistance to humoral immunity. In particular, mutation at residue S477 found in the Omicron variant confers resistance to CPs, while mutation at residue E484 found in Beta, Gamma, and Omicron variants confers resistance to neutralizing monoclonal antibodies (NmAbs), vaccines, and postvaccine sera (12, 14). The spike mutation at residue K417 in almost all VOCs, but not the Alpha variant, has been predicted to cause an overwhelmingly disruptive effect, which may make these variants resistant to vaccine-induced humoral immunity (3, 12). Overall, at the molecular level, these spike mutations induce molecular tridimensional changes at the antibody binding sites, which become inaccessible for antibodies and therefore impede antibody binding (20, 21). Also, the residues changed are such that mutations induce an increased binding affinity (or interaction force) of RBD to angiotensin-converting enzyme 2 (ACE2), like in the cases of mutations V367F, L452Q, N501Y, and D614G, which is associated with increased transmissibility (1, 10, 11, 21–23).

Similarly, antiviral T-cell immunity evasion by VOCs has also been associated with mutations occurring in the SARS-CoV-2 spike protein. More specifically, mutations in several HLA-I-restricted SARS-CoV-2 epitopes were found to effectively allow VOCs, including Alpha, Beta, and Delta, to escape from viral clearance by CD8^+^ cytotoxic T lymphocytes (CD8^+^ CTLs) (24–27). For instance, mutations L452R and Y453F found in B.1.427/429 (also known as CAL.20C) and B1.1.298 variants are associated with resistance to cellular immunity (24). Moreover, infection with SARS-CoV-2 VOCs is followed by a decreased production of IFN-γ and CD8^+^ T-cells and, more interestingly, an almost zero cytotoxic activity of the low titer of CD8^+^ T-cells produced (25, 28). Also, Le Bert et al. (29) found that in SARS-CoV-2 VOC infections, the cytotoxic activity of CD8^+^ T-cells inversely correlates with COVID-19 severity, suggesting that mutations in SARS-CoV-2 S protein may affect the functionality of CD8^+^ T-cell immune response. More specifically, they may probably induce mechanisms inhibiting the cytotoxic activity of CD8^+^ T-cells (25, 26), which therefore allow their over-replication and spread. Unfortunately, unlike the well-documented detrimental effect of mutations on humoral immunity, how the mutations in SARS-CoV-2 VOCs induce T-cell immunity evasion at the molecular level is not well documented.

In this review, we discussed how SARS-CoV-2 VOCs adapt to and escape from anti-COVID-19 cellular immunity by focusing on the effects of specific SARS-CoV-2 mutations on cytotoxic CD8^+^ T-cell immunity activation.

## 2 Activation of CD8^+^ T-cells in viral infections

Most acute respiratory viral infections trigger activation and proliferation of both naïve CD4^+^ and CD8^+^ T-cells, as they play central roles in viral clearance. For instance, mature effector CD8^+^ CTLs are known to block virus multiplication by killing infected cells and secrete antiviral cytokines, including IFN-γ, TNF-α, and infected-cell killer molecules [Fas-L, perforin, and granzyme B (GrB)] (30–32).

The molecular mechanism for activating naïve CD8^+^ T-cells consists of two main pathways, namely thymus-independent (33) and thymus-dependent activation pathways (34, 35). In the thymus-independent activation pathway, CD8^+^ T-cell activation requires virus-infected antigen-presenting cells (APCs), which present a cognate viral peptide to naïve CD8^+^ T-cells. Specifically, following viral entry, the proteasome and other peptidases in the cytosol progressively degrade viral proteins to small specific peptides. The generated peptides are transported into the endoplasmic reticulum (ER) and trimmed by ER aminopeptidase 1, and those with the appropriate/specific motif are loaded onto MHC I molecules. Through the Golgi, peptide-MHC-I molecule complexes transit the plasma membrane and display the loaded viral antigen at the APC surface. Thus, CD8^+^ T-cell activation occurs when the T-cell receptors (TCRs) of CD8^+^ T lymphocytes recognize viral peptides loaded onto MHC I molecules [reviewed in (31)].

Moreover, in the absence of virus-infected APCs displaying their cognate peptide through MHC I molecule binding to naïve TCR CD8^+^ T-cells in secondary lymphoid organs (lymph nodes and spleen), induction of CD8^+^ cytotoxic T lymphocytes may require help from active CD4^+^ T helper cells (31, 34, 35). In this activation pathway, two models have been described: the two- and three-cell models. In the former model, CD4^+^ T-cells first pre-activate APCs such as dendritic cells (DCs) by co-stimulation, which subsequently activate naïve CD8^+^ T-cells. In the later model, both active CD4^+^ Th and naïve CD8^+^ T-cells interact simultaneously with the same APC, and naïve CD8+ T-cell activation occurs through interleukin-2 (IL-2) production by CD4^+^ Th cells (31, 34–37).

After activation, specific mechanisms regulating differentiation and determining the fate of effector CD8^+^ T-cells occur [reviewed in (38)]. Overall, most (but not all) effector CD8^+^ T-cells expand and differentiate into mature effector CTLs to clear viral infections. After viral clearance, the mature effectors that have a shortened lifespan die, while the small remaining set of activated CD8^+^ T-cells differentiates into memory CD8^+^ T-cells, which will help to control secondary infections more efficiently and rapidly (38).

## 3 Cellular immunity in SARS-CoV-2 infection

Studies on T-cell immune responses to SARS-CoV-2 infection are scarce. The substantial role of cellular immunity in SARS-CoV-2 infection has been demonstrated in the few available agammaglobulinemia-related studies, where a standalone T-cell response could complete the viral clearance and assure full recovery in humoral immunodeficiency patients (39–42). Therefore, in COVID-19, like in other respiratory diseases, SARS-CoV-2 infection is followed by a huge and robust immune response mediated by a variety of T-cells, phenotypically and functionally diverse, protecting from severe complications, leading to a quick recovery and conferring long-lasting (memory) immunity.

More specifically, in symptomatic and acute COVID-19 patients, clinical reports have shown a state of characterized lymphopenia, especially in moderate-to-critically ill COVID-19 patients (43–47), in which the T-cell count was lower than that in mild COVID-19 patients and healthy people (normal range 955–2,860 T-cells/µl) (25, 28, 48, 49). Moreover, given that the elderly infected with SARS-CoV-2 have the worst disease outcomes (50), leading to death (51), aged-based studies showed that cellular immune response is reduced, and the T-cell count is far lower in the elderly than that in healthy donors and mild and recovered patients (52). In contrast, in mild COVID-19 patients, a higher T-cell response was observed and characterized in almost all patients (detection of CD4^+^ and CD8^+^ in 80–100% and 70%–80% of COVID-19 patients, respectively [reviewed in (53)], with a higher CD8^+^/CD4^+^ T-cell ratio, along with a higher T-cell count than neutrophils (54, 55). Also, in convalescent antibody-positive and -negative COVID-19 patients, a robust T-cell response was characterized by the presence of reactive CD4^+^CD154^+^CD137^+^ and CD154^+^CD137^+^ T-cells (41). Moreover, other T-cells with activated phenotypes, including CD38^+^, CD39^+^, HLA-DR^+^, Ki-67^+^, and CD69^+^ T-cells, were detected mostly in mild and convalescent COVID-19 patients [reviewed in (53)]. These observations, which positively correlated with the clearance of COVID-19 symptoms and recovery of almost all patients without artificial respiratory assistance, were significantly opposite to those observed in moderate and severe COVID-19 patients (53–55). Thus, it is worthy to conclude that the lymphopenia state positively correlates with COVID-19–associated death (i.e., lymphopenia is a death-determining factor) because people who succumbed to COVID-19 had a significantly lower absolute number of lymphocytes (specifically CD4^+^ and CD8^+^ T-cells) than convalescent patients (56–58). This indicates that COVID-19 patients with a decreased T-cell response, including CD4^+^ and CD8^+^ T-cells, are likely to be more vulnerable to disease severity and fatality, highlighting the central role of CD4^+^ and CD8^+^ T-cells in SARS-CoV-2 clearance. Nevertheless, T-cell exhaustion and dysregulation have been described in COVID-19 [reviewed in (53, 56)], mainly at higher viral loads. However, in immunocompetent patients, this condition may be transient, with the return of CD8^+^ T-cells boosted by effector CD4^+^ and memory CD4^+^ T-cells within 2 to 3 months, as observed in SARS-CoV infections (59).

Furthermore, the diversity of T-cell response has been associated with the production of abundant protective CTL- and Th1-response–inducing cytokines (60). In convalescent mild and severe COVID-19 patients, a high production frequency of double- and triple-positive IFN-γ–, TNF-α–, and IL-2–producing CD4^+^ T-cells has been detected. Also, a similar expression of IFN-γ, TNF-α, GrB, and/or the CD107a marker of degranulation producing CD8^+^ T-cells has been reported (41, 49, 54, 56, 61, 62). In that view, Jordan et al. (63) specified that IL-2 and TNF-α are markers for activated CD4^+^ T-cells and TNF-α and IFN-γ for activated CD8^+^ T-cells. In more severe COVID-19 cases, however, elevated and steady exhaustion levels and reduced functional diversity of T-cells in peripheral blood together with higher production levels of type 2 (IL-5, IL-9, IL-10, and IL-13) and type 3 (IL-17A/F and IL-22) responses have been found, suggesting that this later promotes the activation of the production of proinflammatory cytokines, including IL-1β, IL-6, CXCL8/IL-8, TNF, and CXCL10/IP-10, also associated with neutrophils and lymphoid organ damage (blocking T-cell response) (61, 64, 65), in severe COVID-19 patients.

The detectable reactive T-cell response in COVID-19 patients responsible for the viral clearance has a broad variable specificity to different SARS-CoV-2 proteins. The most dominant reactive T-cells, including CD4^+^, CD8^+^, CD4^+^CD154^+^CD137^+^, and CD154^+^CD137^+^, detected in mild and recovered COVID-19 patients were specific to SARS-CoV-2 structural proteins (SPs), including ORF3a, spike (S), membrane (M), and nucleocapsid (N) (41, 53, 66). Non-structural protein (NSP)-specific T-cells, including SARS-CoV-2 NSP13 of ORF-1, NSP7, and ORF7/8, have also been identified (53, 66, 67).

Moreover, it is important to mention the existence of cross-reactive cellular immunity. Indeed, several reports demonstrated a preexisting protective T-cell immunity against COVID-19, specific to SP and NSP from human coronaviruses (hCoVs) other than SARS-CoV-2, in healthy and SARS-CoV-2 non-exposed adults and in blood samples obtained before the COVID-19 outbreak. Similarly, SARS-CoV-2–specific T-cell response is found to cross-react with other HCoV proteins (41, 66). This suggests that, similar to SARS-CoV–specific T-cell response, which displays a robust cross-reactivity to SARS-CoV-2 proteins after 17 years post-infection, SARS-CoV-2–specific T-cell immunity may persist in recovered COVID-19 patients, allowing for rapid clearance of the infection in the case of secondary infection with SARS-CoV-2 (66) and—probably—SARS-CoV-2 variants, but not all (68). For instance, some studies reported that the reinfection rate by WT SARS-CoV-2 was very low (absolute rate of 0%–1.1%) in individuals who recovered from WT SARS-CoV-2 infection, and their immune responses were elevated and steady for at least 10 months (68, 69). Note that this estimated law reinfection rate was related to reinfection by the same WT SARS-CoV-2. The low reinfection rate by VOCs due to cross-protection by SARS-CoV-2 T-cell immunity remains speculative and confirmed, even though the preexisting WT SARS-CoV-2 cellular immunity may contribute to the attenuation of VOC-associated clinical severity (68).

In contrast, current newborns and children are unlikely to have preexisting cross-reactive T-cell immunity against SARS-CoV-2, as they have not been exposed to SARS, MERS, and/or other circulating HCoVs. This is supported by Cohen et al. (70), who demonstrated that memory CD4^+^ T-cell response increases with age, and CD8^+^ T-cell response increases with time post-infection, explaining the significantly lower SARS-CoV-2 T-cell response and preexisting cross-reactive CD4^+^ and specifically CD8^+^ T-cell immunity against SARS-CoV-2 in children and newborns than in adults (70). This suggests that CD8^+^ T-cell immunity will take longer to maturate and clear SARS-CoV-2 infection in infants than in adults.

## 4 VOCs evade CD8^+^ T-cell immunity and adapt to host cells

In a recent study, Alison et al. (71) demonstrated that WT SARS-CoV-2–specific T-cell natural and vaccine-induced immunity is not negatively or is lightly affected by but could still recognize VOCs, including Alpha (B.1.1.7), Beta (B.1.351), Gamma (P.1), and CAL.20C variants, and that only 7% and 3% of CD4^+^ and CD8^+^ T-cell epitopes are mutated, respectively. Mazzoni et al. (72) also supported and specified in their study that the WT SARS-CoV-2–specific CD4^+^ T-cell response is more conserved against VOCs because mutations mainly occur within non-CD4^+^ T-cell epitopes, which might suggest that clearance of VOC infection could be mediated mainly by preexisting SARS-CoV-2–specific CD4^+^ T-cells. This allows them and some other scientists (73) to hypothesize that despite mutations in T-cell epitopes and because of the broad conserved T-cell epitope coverage, WT SARS-CoV-2–specific T-cell immune response (regardless of the immunity-mediating T-cell types) may still contribute to reducing SARS-CoV-2 (including WT and VOCs) infection severity.

However, mutations in 3% of CD8^+^ T-cell epitopes make a huge difference. They may lead to indescribable fatalities due to more virulent mutants, as reported by Elisa Guo and Hailong Guo (74). They found that “CD8^+^ T-cell epitope mutants of SARS-CoV-2 proteins lead to persistently variable SARS-CoV-2 infections with different susceptibility and severity” (74, 75). Indeed, several other studies demonstrated with solid evidence that, despite the preexisting SARS-CoV-2–specific cellular immunity in COVID-19 recovered patients, the viral replication rate after reinfection with SARS-CoV-2, but specifically SARS-CoV-2 VOCs, is increased in these patients (76). More importantly, although the presence of CD8^+^ T-cell immune response against VOCs in WT COVID-19 convalescent or recovered patients was reported, as claimed previously (71, 72), these CD8^+^ CTLs were non-functional or ineffective against VOCs (76–78). Gallagher et al. also demonstrated that VOCs escape from vaccine CD8^+^ T-cell immune response as they found a decreased T-cell immunity against VOCs (Alpha (B.1.1.7), Beta (B.1.351), and B.1.1.248 variants) in patients vaccinated with specific SARS-CoV-2 mRNA vaccines from Moderna and Pfizer compared with T-cell responses to WT SARS-CoV-2 infection (79). These clinical features in COVID-19 suggest that preexisting SARS-CoV-2–specific T-cell responses might be ineffective against infection with VOCs and imply that SARS-CoV-2, but more probably VOCs, can still escape from CD8^+^ T-cell immunity and lead to inactivation of T-cell immunity while maintaining active viral replication (80–83). This is the main clinical characteristic of the Omicron variant, mainly described as mild symptomatic infection, with an increased infection rate in SARS-CoV-2 recovered patients (83, 84).

Furthermore, compared with CD4^+^ T-cell epitopes, CD8^+^ T-cell epitopes are more vulnerable. Indeed, CD8^+^ T-cell HLA-I epitopes are shorter (8 to 10 residues) than CD4^+^ T-cell HLA-II epitopes (12 to 16 residues). A single mutation in one of the CD8^+^ T-cell HLA epitopes is enough and sufficient to impair and compromise recognition of epitopes by HLA, thus inhibiting activation, functionality, and cytotoxic activity of CD8^+^ T-cells, which considerably and specifically inhibits the destruction of infected host cells (62, 75) and generally affects the overall T-cell response efficacy. Understandably, subversion of CD8^+^ T-cell response affects the potency of the whole T-cell response because, in the context of the SARS-CoV-2 threat, the viral replication mechanism is exclusively intracellular, and the main involved T-cell response is led by CD8^+^ CTLs, due to efficient presentation of endogenously produced antigens on MHC-I molecules. Pretti et al. (85) demonstrated in an *in silico* analysis of VOCs’ epitopes of CD8^+^ T-cells that a single mutation including E484K in spike protein may induce T-cell evasion as it alters the binding of the peptide onto its corresponding HLA of MHC-I (**Table 1**). More interestingly, it has been shown that non-functional and/or exhaustion of CD8^+^ T-cells in convalescent non-human primates significantly decreases the protective efficacy of natural immunity against SARS-CoV-2 and promotes infectivity and severity of SARS-CoV-2 VOCs. Also, in critically ill COVID-19 patients, a lower CD8^+^/CD4^+^ T-cell ratio was discovered (i.e., a low titer of CD8^+^ T-cells), suggesting that functional CD8^+^ T-cells, but better associated with CD4^+^ T-cells in SARS-CoV-2 infection, are therefore required for preventing infection severity associated with a better viral clearance (24, 25, 28, 29, 53, 88).

**Table 1:** The signature mutations of VOCs and mechanism of immune escape.

SARS-CoV-2 VOCs	Pango lineages^a^	Mutations in spike^b^	Mechanism of escape/resistance cell immunity (CD8^+^ T-cells)^d^
Alpha	B.1.1.7	**Q27 stop** ^c^, **Δ69-70**, Δ144, **N501Y**, A570D, D614G, P681H, T716I, S982A, D1118H	ORF8 truncation enhances the downregulation of MHC-I through the lysosomal autophagy pathway (86)
Beta	B.1.351 B.1.351.2 B.1.351.3	L18F, D80A, D215G, Δ242-244, R246I, **K417N** ^c^, **E484K** ^c^, **N501Y**, D614G, A701V	Variant epitope reduces HLA-I-peptide-binding affinity and inhibits activation of CD8^+^ T-cells (85, 87)
Gamma	P.1 P.1.1 P.1.2	L18F, T20N, P26S, D138Y, R190S, **K417T** ^c^, **E484K** ^c^, **N501Y**, D614G, H655Y, T1027I, V1176F	Variant epitope reduces HLA-I-peptide-binding affinity and inhibits activation of CD8^+^ T-cells (87)
Delta	B.1.617.2	T19R, Δ157-158, **L452R** ^c^, **T478K**, D614G, P681R, D950N	Variant epitope reduces HLA-I-peptide-binding affinity and inhibits activation of CD8^+^ T-cells (87) or resists pre-cell immunity (24)
Kappa	B.1.617.1	G142D, E154K, **L452R** ^c^, **E484Q** ^c^, D614G, P681R, Q1071H	Variant epitope significantly reduces the ability to activate CD8^+^ T-cells through loss of affinity to HLA-I molecules (85, 87) or resists pre-cell immunity (24)
	C.1.2	C136F, Y144del, R190S, D215G, LA242-243del, **Y449H**, **E484K** ^c^, **N501Y**, N679K, T716I, P9L, D614G, H655Y, T859N	Variant epitope reduces HLA-I-peptide-binding affinity and inhibits activation of CD8^+^ T-cells (87)
Omicron^b^	B.1.1.529	A67V, **Δ69-70**, T95I, G142D, Δ143-145, Δ211, L212I, ins214EPE, **G339D**, S371L, **S373P**, **S375F**, **K417N** ^c^, **N440K**, **G446S**, **S477N**, **T478K**, **E484A** ^c^, **Q493R**, **G496S**, **Q498R**, **N501Y**, **Y505H**, **T547K**, D614G, H655Y, N679K, P681H, N764K, D796Y, N856K, Q954H, N969K, L981F	Variant epitope reduces HLA-I-binding peptide affinity and inhibits activation of CD8^+^ T-cells (85, 87)

VOCs, variants of concern.

a Phylogenetic Assignment of Named Global Outbreak (Pango) Lineages is a dynamic nomenclature using the PANGOLIN computational system to classify genetic lineages for SARS-CoV-2 and its relative variants (6-9).

b https://covdb.stanford.edu/page/mutation-viewer/#sec_alpha.

c Signature mutation in VOCs that are revealed to induce CD8^+^ T-cell immune escape, so far. In **bold**, mutations that enhance infectivity, severity, and immune escape by VOCs.

d The described mechanism of escape/resistance cell immunity is related to mutations in **bold**.

Prior-to-SARS-CoV-2 outbreak studies demonstrated that antiviral cellular immunity evasion by variants is associated with mutations occurring in CTL epitopes (involved in T-cell activation), which results in enhanced infection severity (89, 90) (**Tables 1**, **2**). Similarly, recent studies corroborate these previous findings, demonstrating that in infections with emerging SARS-CoV-2 VOCs, there is low production of IFN-γ and CD8^+^ T-cells and an almost zero cytotoxic activity of the latter (25). Specifically, they demonstrated that non-synonymous single mutations of CD8^+^ T-cell epitopes found in most VOCs induce inhibition of MHC-I binding in a cell-free *in vitro* assay, resulting in reduced and non-functional CD8^+^ T-cell production (25, 26), which demonstrated that mutations in VOCs evade CD8^+^ T-cell immunity and adapt into host cells (**Table 1**). The same results were found by Motozono et al. (24), describing a reduced potency of CTL, followed by increased COVID-19 infectivity and severity, in SARS-CoV-2 VOC-infected people. Given the demonstrated negative effect of SARS-CoV-2 mutants on the functionality of CD8^+^ T-cell immune responses, potential mechanisms underlying these effects must be documented.

**Table 2:** Dominant and strictly validated non-conserved CD8^+^ T-cell–activating epitopes of SARS-CoV-2 involved in VOC immune escape.

Protein	A_1_ ^a^	Epitope sequence	Mutation(s)/mutants	HLA-I genotype	(Ref)
M	61	TLACFVLAAV	TLACFVLA**V**V	HLA-A*02:01	(25)
M	61	TLACFVLAAV	TLACFV**P**AAV	HLA-A*02:01	(25)
M	61	TLACFVLAAV	**I**LACFVLAAV	HLA-A*02:01	(25)
M	61	TLACFVLAAV	TLACFVLAA**F**	HLA-A*02:01	(25)
M	61	TLACFVLAAV	TLA**F**FVLAAV	HLA-A*02:01	(25)
M	61	TLACFVLAAV	TLACFVLAVV	HLA-A*02:01	(25)
M	61	TLACFVLAAV	TLACFVL**S**AV	HLA-A*02:01	(25)
M	61	TLACFVLAAV	TLA**Y**FVLAAV	HLA-A*02:01	(25)
M	61	TLACFVLAAV	ILACFVLAAV	HLA-A*02:01	(25)
M	61	TLACFVLAAV	TL**V**CFVLAAV	HLA-A*02:01	(25)
M	61	TLACFVLAAV	ILACFVLAAV	HLA-A*02:01	(25)
M	61	TLACFVLAAV	TLACFVLAVV	HLA-A*02:01	(25)
M	61	TLACFVLAAV	TLACFVLA**P**V	HLA-A*02:01	(25)
M	61	TLACFVLAAV	TLACFVLA**S**V	HLA-A*02:01	(25)
M	89	GLMWLSYFI	G**F**MWLSYFI	HLA-A*02:01	(25)
M	89	GLMWLSYFI	GLMWL**I**YFI	HLA-A*02:01	(25)
M	89	GLMWLSYFI	GLM**C**LSYFI	HLA-A*02:01	(25)
M	89	GLMWLSYFI	**C**LMWLSYFI	HLA-A*02:01	(25)
M	89	GLMWLSYFI	GL**I**WLSYFI	HLA-A*02:01	(25)
M	89	GLMWLSYFI	GLM**R**LSYFI	HLA-A*02:01	(25)
M	89	GLMWLSYFI	GLMWL**T**YFI	HLA-A*02:01	(25)
N	322	MEVTPSGTWL	MEVTP**L**GTWL	HLA-B*40:01	(25)
N	322	MEVTPSGTWL	MEV**I**PSGTWL	HLA-B*40:01	(25)
N	322	MEVTPSGTWL	MEVTPSGTW**F**	HLA-B*40:01	(25)
N	322	MEVTPSGTWL	**I**EVTPSGTWL	HLA-B*40:01	(25)
N	322	MEVTPSGTWL	MEVTPSGTW**S**	HLA-B*40:01	(25)
N	322	MEVTPSGTWL	ME**A**TPSGTWL	HLA-B*40:01	(25)
N	322	MEVTPSGTWL	MEVT**L**SGTWL	HLA-B*40:01	(25)
N	322	MEVTPSGTWL	**V**EVTPSGTWL	HLA-B*40:01	(25)
NSP2	461	FLRDGWEIV		HLA-A*02:01	(91)
NSP2	85	TFNGECPNF		HLA-A*24:02	(92)
nsp3	364	LYDKLVSSF		HLA-A*24:02	(92)
nsp3	1,081	YYKKDNSYF		HLA-A*24:02	(92)
nsp3	1,512	AYILFTRFF		HLA-A*24:02	(92)
Nsp4	486	LYQPPQTSI	LYQPPQ**I**7SI	HLA-A*24:02	(92)
Nsp5	140	FLNGSCGSV^c^		HLA-A*02:01	(91)
Nsp5	204	VLAWLYAAV^c^		HLA-A*02:01	(91)
Nsp7	27	KLWAQCVQL		HLA-A*02:01	(91)
ORF1a	2,230	IIWFLLLSV	**T**IWFLLLSV	HLA-A*02:01	(87)
S	2	FVFLVLLPLV^b^	FVFLVL**V**PLV	HLA-A*02:01	(75)
S	2	FVFLVLLPLV^b^	FVF**F**VLLPLV^b^	HLA-A*02:01	(75)
S	2	FVFLVLLPLV^b^	FVFLVLL**S**LV	HLA-A*02:01	(75)
S	2	FVFLVLLPLV^b^	FVFLVLL**L**LV	HLA-A*02:01	(75)
S	2	FVFLVLLPLV^b^	FVFLVL**W**PLV	HLA-A*02:01	(75)
S	2	FVFLVLLPLV^b^	FVFLVLL**T**LV	HLA-A*02:01	(75)
S	2	FVFLVLLPLV^b^	FVFLVLL**Q**LV	HLA-A*02:01	(75)
S	2	FVFLVLLPLV^b^	FVF**I**VLLPLV^b^	HLA-A*02:01	(75)
S	2	FVFLVLLPLV^b^	FVF**F**VLL**S**LV^b^	HLA-A*02:01	(75)
S	2	FVFLVLLPLV^b^	FVF**F**VL**F**PLV^b^	HLA-A*02:01	(75)
S	133	FQFCNDPFL^b^	FQFCN**Y**PFL^b^	HLA-A*02:01	(75)
S	133	FQFCNDPFL^b^	FQFCN**H**PFL^b^	HLA-A*02:01	(75)
S	612	YQDVNCTEV^b^	YQ**G**VNCTEV^b^	HLA-A*02:01	(75)
S	612	YQDVNCTEV^b^	YQ**N**VNCTEV	HLA-A*02:01	(75)
S	612	YQDVNCTEV^b^	YQ**S**VNCTEV	HLA-A*02:01	(75)
S	612	YQDVNCTEV^b^	YQ**A**VNCTEV	HLA-A*02:01	(75)
S	417	KIADYNYKL	**T**IADYNYKL	HLA-A*02:01	(87)
S	417	KIADYNYKL	**N**IADYNYKL	HLA-A*02:01	(25, 87)
S	417	KIADYNYKL	KI**V**DYNYKL	HLA-A*02:01	(25)
S	417	KIADYNYKL	KIAD**N**NYKL	HLA-A*02:01	(25)
S	417	KIADYNYKL	**R**IADYNYKL	HLA-A*02:01	(25)
S	424	KLPDDFTGCV	KLPDDFTG**C**V	HLA-A*02:01	(25)
S	424	KLPDDFTGCV	KLP**Y**DFTGCV	HLA-A*02:01	(25)
S	424	KLPDDFTGCV	KLPDDF**I**GCV	HLA-A*02:01	(25)
S	424	KLPDDFTGCV	KLPD**E**FTGCV	HLA-A*02:01	(25)
S	424	KLPDDFTGCV	KLPDDFTG**F**V	HLA-A*02:01	(25)
S	424	KLPDDFTGCV	KLP**E**DFTGCV	HLA-A*02:01	(25)
S	424	KLPDDFTGCV	KLPD**N**FTGCV	HLA-A*02:01	(25)
S	424	KLPDDFTGCV	KLPD**H**FTGCV	HLA-A*02:01	(25)
S	424	KLPDDFTGCV	KLPDDFTGC**F**	HLA-A*02:01	(25)
S	424	KLPDDFTGCV	KL**S**DDFTGCV^b^	HLA-A*02:01	(25)
S	821	LLFNKVTLA	L**F**FNKVTLA	HLA-A*02:01	(25)
S	821	LLFNKVTLA	LLFNKV**R**LA	HLA-A*02:01	(25)
S	821	LLFNKVTLA	L**P**FNKVTLA	HLA-A*02:01	(25)
S	821	LLFNKVTLA	LLFNK**L**TLA	HLA-A*02:01	(25)
S	821	LLFNKVTLA	LLFNK**A**TLA	HLA-A*02:01	(25)
S	821	LLFNKVTLA	**P**LFNKVTLA	HLA-A*02:01	(25)
S	821	LLFNKVTLA	LLFNKVTL**T**	HLA-A*02:01	(25)
S	1,185	RLNEVAKNL	RLNEVA**N**NL	HLA-A*02:01	(25)
S	1,185	RLNEVAKNL	RLNEV**S**KNL	HLA-A*02:01	(25)
S	1,185	RLNEVAKNL	RLNEV**V**KNL	HLA-A*02:01	(25)
S	1,185	RLNEVAKNL	**C**LNEVAKNL	HLA-A*02:01	(25)
S	1,185	RLNEVAKNL	R**F**NEVAKNL	HLA-A*02:01	(25)
S	1,185	RLNEVAKNL	RLNE**A**AKNL	HLA-A*02:01	(25)
S	1,185	RLNEVAKNL	RLNEVAK**I**L	HLA-A*02:01	(25)
S	1,185	RLNEVAKNL	RL**T**EVAKNL	HLA-A*02:01	(25)
S	1,185	RLNEVAKNL	RLNEVAKN**S**	HLA-A*02:01	(25)
S	1,185	RLNEVAKNL	RLNEVA**T**NL	HLA-A*02:01	(25)
S	1,185	RLNEVAKNL	RLNE**A**AKNL	HLA-A*02:01	(25)
S	269	YLQPRTFLL	Y**F**QPRTFLL	HLA-A*02:01	(25, 27)
S	269	YLQPRTFLL	YLQPR**I**FLL *^b^*	HLA-A*02:01	(25)
S	269	YLQPRTFLL	YLQPRTFL**F**	HLA-A*02:01	(25)
S	691	SIIAYTMSL	**F**IIAYTMSL	HLA-A*02:01	(25)
S	691	SIIAYTMSL	**C**IIAYTMSL	HLA-A*02:01	(25)
S	691	SIIAYTMSL	S**T**IAYTMSL	HLA-A*02:01	(25)
S	691	SIIAYTMSL	SII**P**YTMSL	HLA-A*02:01	(25)
S	691	SIIAYTMSL	SIIAYTM**L**L	HLA-A*02:01	(25)
S	144	GVYYHKNNK	GVY**-**HKNNK	HLA-A*11:01	(87)
S	448	NYNYLYRLF	NYNY**R**YRLF^b^	HLA-A*24:02	(24, 87)
S	448	NYNYLYRLF	NYNYL**F**RLF^b^	HLA-A*24:02	(24)
S	269	YLQPRTFLL^b^	YLQ**L**RTFLL	HLA-A*02:01	(27)
S	1,000	RLQSLQTYV^b^	RLQSLQ**I**YV	HLA-A*02:01	(27)
S	1,000	RLQSLQTYV^b^	RLQSL**H**TYV^b^	HLA-A*02:01	(25)

a Position of the first residue of CD8^+^ T-cell epitope sequence.

b Dominant epitopes with the highest mutation rates and associated with a decreased recognition by WT SARS-CoV-2–specific CD8^+^ T-cell immunity (driving the immune escape).

c Dominant epitopes with the lowest mutation rates. In bold and red, dominant mutation reported in SARS-CoV-2 variants of concern.

## 5 Mechanisms of CD8^+^ T-cell immune escape by SARS-CoV-2 VOCs

In general, viral replication is a natural survival process that viruses go through and which unfortunately causes damage to their hosts, which, in turn, counterattacks to eliminate the viral infection *via* a protective immune response. To escape the host immunity, especially the cellular but CD8^+^ T-cell immune response, in COVID-19, SARS-CoV-2 uses certain evasion mechanisms, including genomic changes, under the host immune pressure, which yield variants with selective and survival advantages and enhanced viral fitness. These are literally followed by increased infectivity and severity. These modifications include up- or downregulation of certain viral gene expression mechanisms or non-synonymous mutations in gene sequences involved in immune response activation.

### 5.1 SARS-CoV-2 VOCs enhance MHC-I degradation through its ORF8 protein

The SARS-CoV-2 ORF8 protein is 121 amino acids long and consists of a covalent disulfide-linked dimer formed through the N-terminal sequence and a separate non-covalent interface formed by _73_YIDI_76_, another SARS-CoV-2–specific sequence. Moreover, the ORF8 protein N-terminal sequence is followed by an Ig-like fold and a signal peptide for endoplasmic reticulum (ER) entry, where ORF8 protein interacts with host proteins, including factors involved in ER-associated degradation (93, 94).

**Figure 1:**
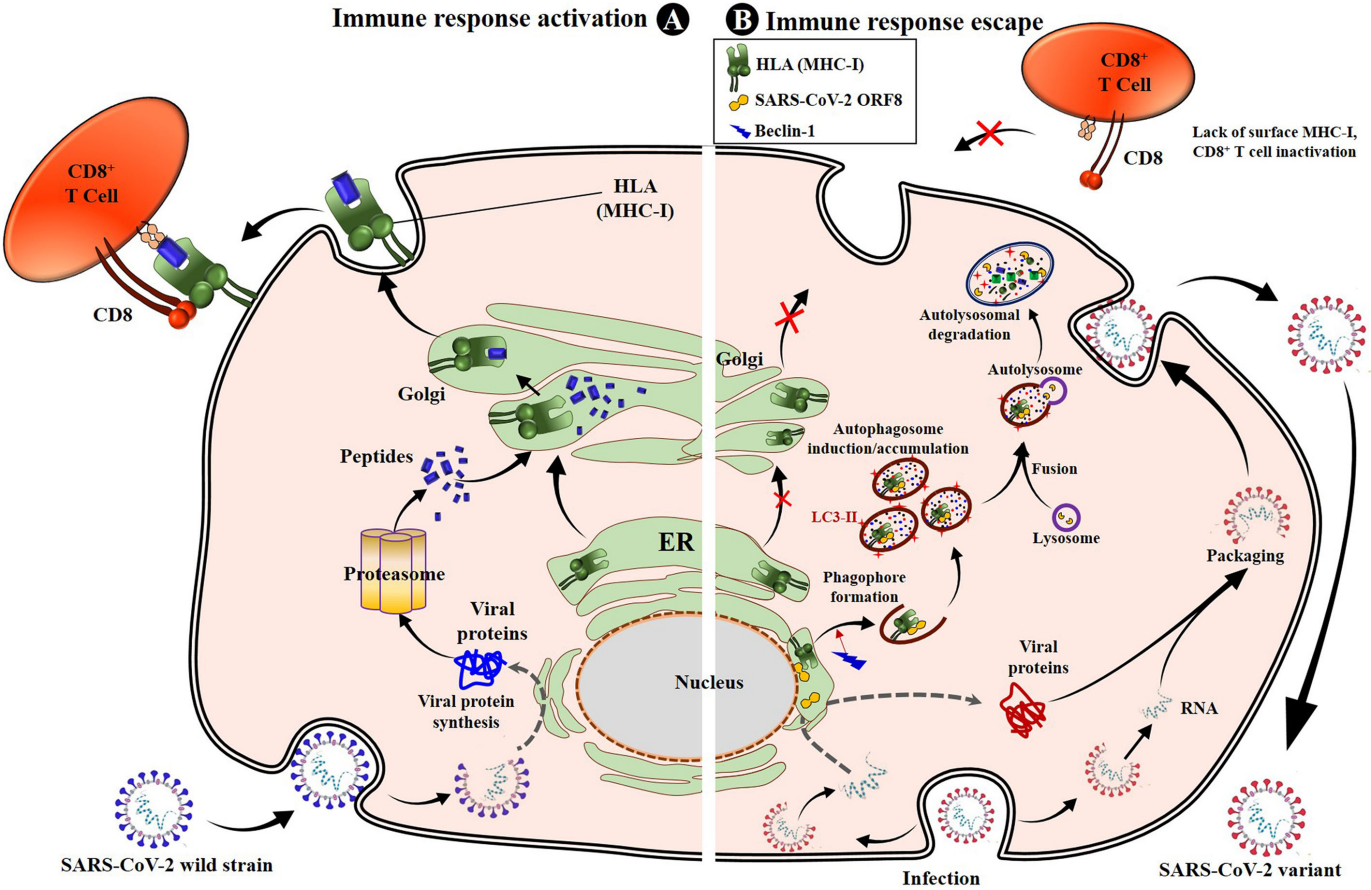
SARS-CoV-2 impairs antigen presentation by MHC-I to CD8+ T cells through ORF8. Once entered into the epithelial cells by endocytosis, the genomic RNA is released. SARS-CoV-2 uses the cell protein expression machinery to synthesize the viral proteins. The antigen processing and presentation pathways occur for viral protein lysis into peptides. Simultaneously, the MHC-I will mature in the ER and migrate to the Golgi apparatus, where peptides will be loaded onto MHC-I molecules and presented to CD8^+^ cytotoxic T lymphocytes (CTLs), activating the cell cytotoxic response **(A)**. When infected with SARS-CoV-2, especially variants of concern, the viral proteins, including the ORF8, are produced inside ER-derived DMVs containing LC3-I. The synthesized ORF8 protein directly interacts with the MHC-I and leads MHC-I trafficking from ER to autophagosome vesicles, inducing the early stages of autophagy and accumulation of autophagosomes thanks to beclin 1-activated upregulation. The matured autophagosome then fuses with the lysosome to form the autolysosome, inside which MHC-I is digested by lysozymes. This results in the loss of sensitivity of SARS-CoV-2–infected cells to CD8^+^ T cells and lysis by CTLs. When infection occurs with VOCs, the mechanism is strongly enhanced, and the SARS-CoV-2 variant easily escapes T cells **(B)**. ER, endoplasmic reticulum; DMV, doubled membrane vesicle; LC3-I, microtubule-associated protein 1A/1B-light chain 3B; MHC-I, major histocompatibility complex type I.

It has been found that SARS-CoV-2 uses the product of its ORF8 gene to escape CD8^+^ T-cell immunity through disruption or a downregulation of the mechanism of antigen presentation to CD8^+^ T-cells by the MHC-I (82). Specifically, the ORF8 protein of SARS-CoV-2 directly interacts with the MHC-I molecules and strictly induces their downregulation. The direct interaction occurs in the ER, and once the complex ORF8-MHC-I molecule is formed, the ORF8 product induces MHC-I trafficking from the ER to lysosomes mediated by ER-phagy for lysosomal vesicle degradation by autophagy. It is, in fact, the subsequent interaction of ORF8 protein with Beclin 1 [a key molecule in autophagy initiation (95)] that induces activation of the autophagy pathway and the further degradation of MHC-I, which is responsible for the lower sensitivity of SARS-CoV-2–infected cells to lysis by CTLs (82) (**Figure 1**). This evasion mechanism is enhanced in infections by VOCs (82, 96, 97). Indeed, mutations in the ORF8 gene have been associated with increased severity, transmissibility, and especially immune evasion (86, 94, 96, 98). Specifically, many reports have identified non-synonymous mutations or truncations in the ORF8 gene of VOCs (86, 96), explaining in part the enhanced immune escape by these VOCs, including the variant Alpha (202012/01 or B.1.1.7), which has a mutation (Q27 stop codon) that truncates ORF8 (86). Therefore, these SARS-CoV-2 VOCs use their selective ORF8 mutant proteins to enhance the above-described mechanism of activation of the autophagy pathway and the lysosomal degradation of MHC-I, which yields an increased inactivation of the CTL response (**Figure 1**). Fortunately, experiments have demonstrated that a knockdown or a complete deletion of ORF8 activates surface MHC-I proper expression and significantly reduces immune escape (82, 96), suggesting that inhibiting ORF8 of SARS-CoV-2 by some specific body-harmless nanoparticles or nanobodies (82) constitutes a way to alleviate immune escape by VOCs and enhance CD8^+^ T-cell efficacy.

### 5.2 SARS-CoV-2 VOCs abolish CTL response activation through CD8^+^ T-cell epitope mutations

#### 5.2.1 Mutations impair epitope loading onto HLA molecules

**Figure 2:**
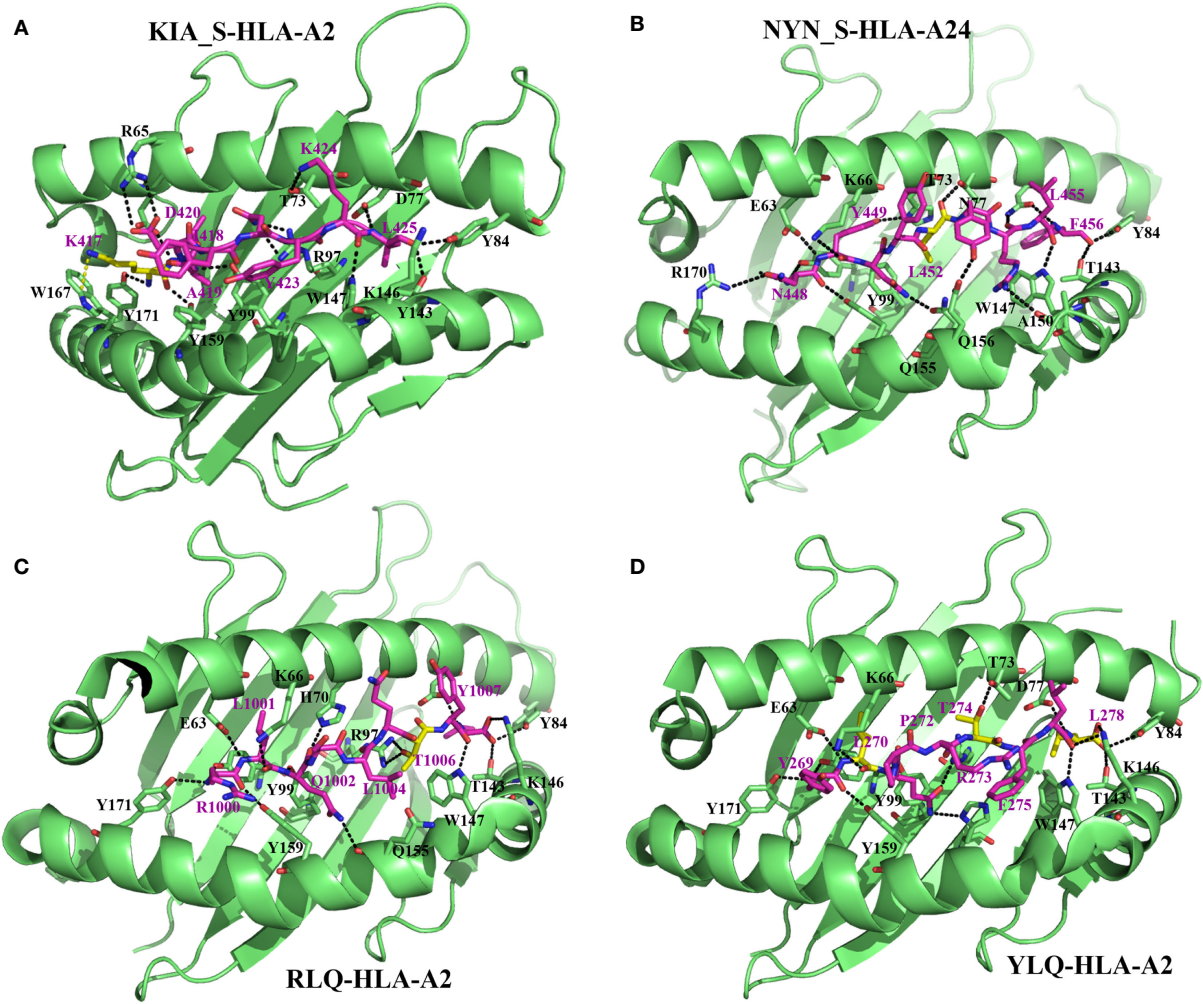
Molecular bases of epitope–HLA-I complexes and effects of epitope mutations. Interactions between KIA_S peptide and HLA-A*02:01 (from PDB:7EU2) **(A)**, NYN_S peptide and HLA-A*24:02 (7F4W) **(B)**, RLQ peptide and HLA-A*02:01 (7N1B) **(C)**, and YLQ peptide and HLA-A*02:01 (7N1A) **(D)**. For all the structures, HLA heavy chains are green, the SARS-CoV-2 peptides are purple, and residues with predominant mutational rates with an effect on immune escape are shown in yellow. Residues at the interface of the interaction of HLA with peptides are represented: nitrogen atoms in blue, oxygen atoms in red, and hydrogen bonds are indicated by black dashed lines.

**Figure 3:**
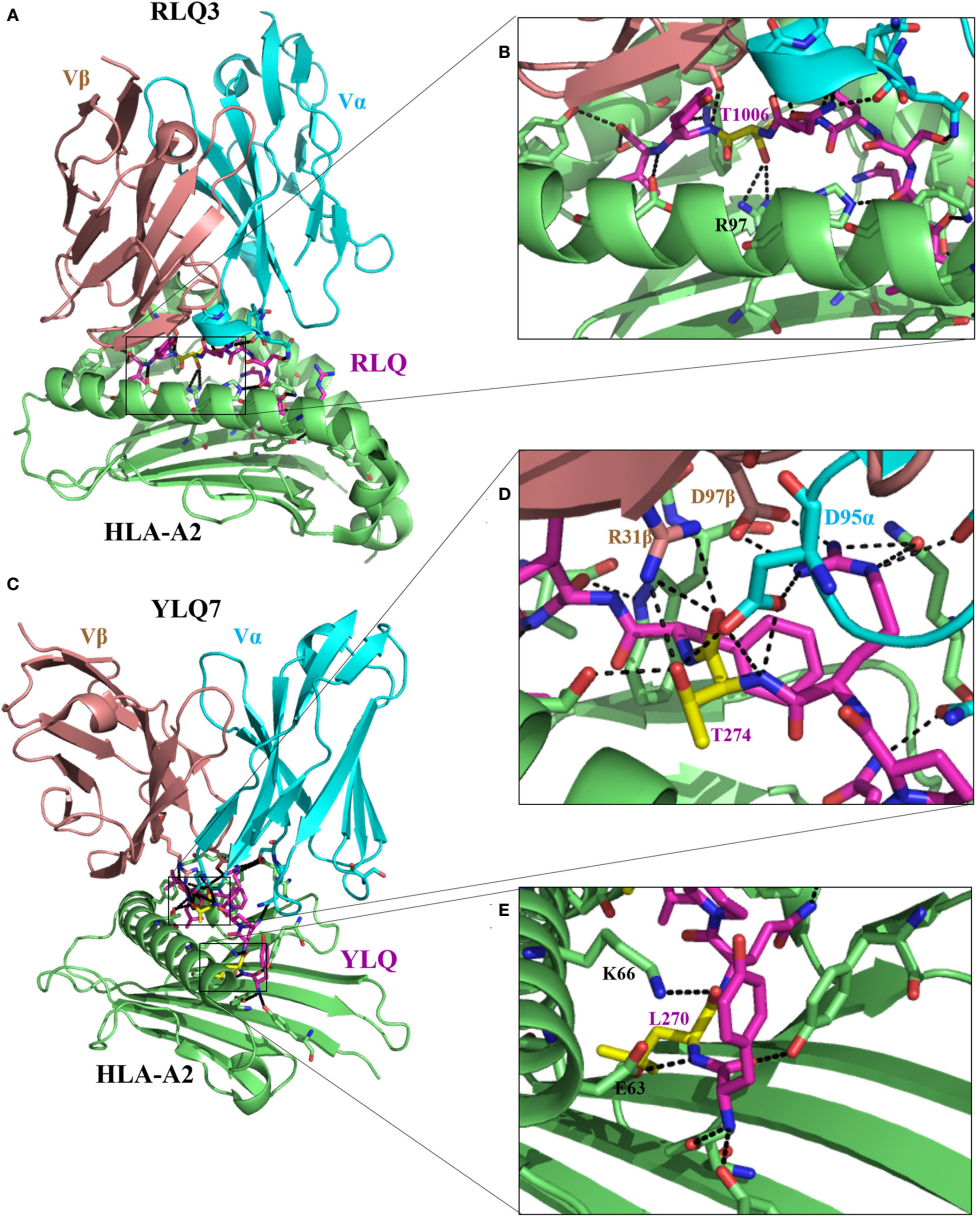
Molecular bases of TCR–epitope–HLA-I complexes and effects of epitope mutations. **(A)** Overview of RLQ3–RLQ–HLA-A2 complex (7N1E). **(B)** Close-up view of interactions of T1006 (yellow), residue with predominant mutational rates in VOCs, with TCR RLQ3 and HLA-A2. **(C)** Overview of YLQ7–YLQ–HLA-A2 complex (7N1F). **(D, E)** Close-up view of interactions of T274 and L270 (yellow), with TCR YLQ7 and HLA-A2. For all the structures, HLA heavy chains are green, the SARS-CoV-2 peptides are purple, while residues with a predominant mutational rate with an effect on immune escape are shown in yellow. Residues at the interface of the interaction of HLA with peptides are represented: nitrogen atoms in blue, oxygen atoms in red, and hydrogen bonds are indicated by black dashed lines.

**Figure 4:**
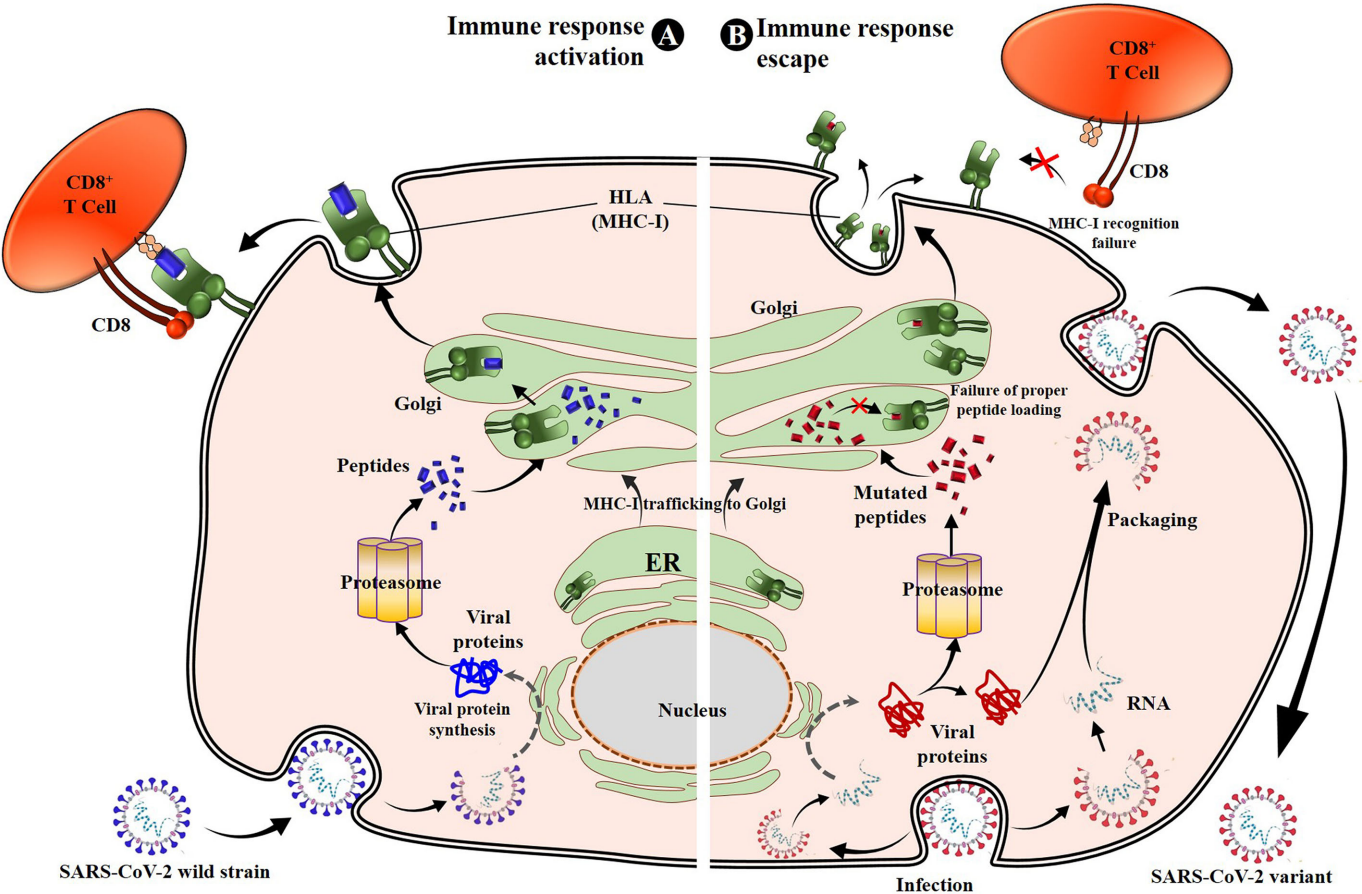
SARS-CoV-2 S protein mutations prevent cellular immunity activation. Once entered into the target cell, SARS-CoV-2 releases its genomic RNA, which serves to produce the viral proteins, including structural and nonstructural proteins. Recognized as non-self-molecules, antigen processing and presentation pathways occur for viral protein lysis into peptides that are then loaded onto the MHC-I or HLA molecules and presented to CD8^+^ CTLs, activating the cell cytotoxic response. The CD8^+^ T cells produce numerous toxic substances (perforins, granzyme, and FasL) and cytokines (IFN-γ, TNF-α, and IL-2) directly involved in SARS-CoV-2–infected cell death **(A)** (30, 32). However, because of the mutations in specific antigen peptides (such as spike-mutated derived antigens), these later lose their binding affinity to HLA-I. Consequently, the peptides are either not loaded or unstably loaded onto the corresponding HLA molecules. This leads to the reduction or non-activation of the CD8^+^ T cells through low affinity or absence of HLA-I-peptide recognition by TCR, resulting in cellular immune escape and infection maintenance by SARS-CoV-2 variants **(B)**. MHC-I, major histocompatibility complex type I; CTL, cytotoxic T lymphocyte.

Numerous reports demonstrate that SARS-CoV-2 uses mutation-based strategies to downregulate activation pathways of CD8^+^ T response and evade viral clearance. Thus, despite the high rate of conserved T-cell epitopes in SARS-CoV-2 mutants (71, 72), any changes occurring in dominant CD8^+^ CTL epitopes involved in the activation of the T-cell immune response have a negative effect on CD8^+^ T-cell activation, specifically causing deficiency of antigen HLA-A binding and CD8^+^ CTL activation (75, 89, 90) (**Figures 2**, **3**; **Table 2**). Pretti et al. (85) demonstrated that in an *in silico* analysis of VOCs’ epitopes of CD8^+^ T-cells, a single mutation including E484K in spike protein induces T-cell evasion as it alters the binding of the peptide onto its corresponding HLA molecules of MHC-I. Qiu et al. (75) also demonstrated that, while dominant CD8^+^ T-cell epitopes including n-Sp1 of SARS-CoV-2 induce epitope-specific T-cell responses with cytolytic activity toward target cells through HLA-A*02:01 binding, mutations in these epitopes cause potential peptide–HLA-A2 binding deficiency and a decreased CTL activation (**Figures 2**–**4**). Specifically, of the 15 predicted HLA-A*02:01-restricted peptides of S protein, 13 peptides could bind to HLA-A*02:01, while tetramers from seven peptides (n-Sp1, n-Sp2, n-Sp6, n-Sp7, n-Sp11, n-Sp13, and n-Sp14) could detect antigen-specific CD8+ T-cells in COVID-19 convalescent patients and activate CD8^+^ T-cell immunity. Subsequent analyses demonstrated that these seven antigen peptides are the least conserved in SARS-CoV-2 variants, bearing 19, 9, 13, 10, 12, 10, and 9 types of variations, respectively, and that these variant peptides hamper the HLA molecule binding and significantly reduce MHC-I antigen presentation and thus CD8^+^ T-cell activation. This suggests that mutations occur in high frequency in around 50% of CD8^+^ T-cell epitopes (7/14), reducing CD8^+^ T-cell activation by half.

From a molecular point of view, Zhang et al. (87) recently solved crystal structures of two novel crucial CD8^+^ T-cell epitopes of SARS-CoV-2 (KIA_S and NYN_S) involved in cellular immunity activation in complex with their HLA molecule receptors (HLA-A*02:01 and HLA-A*24:02, respectively). They showed that KIA_S and NYN_S peptides specifically form strong and stable complexes with HLA-A*02:01 and HLA-A*24:02, respectively (**Figures 2A, B**), which aligns with their respective ability to activate CD8^+^ T-cell immunity. However, non-synonymous substitutions of residues K417 (from KIA_S) (**Figure 2A**) and L452 (from NYN_S) (**Figure 2B**), which are not conserved in either of the three VOCs (B.1.1.7, B.1.351, or P.1), lead to the loss of affinity of the two mutant peptides to their specific relevant HLA and significantly induce relative VOCs to prevent the activation of and escape from CD8^+^ CTL responses (87). More specifically, in the KIA_S/HLA-A*02:01 complex, the cation–pi interaction (K417–W167 bound) is the main bond that stabilizes the complex (**Figure 2A**) (87) over others (salt bridge interactions), which are weakened due to the acidic environment in the Golgi (99). In VOCs, including B.1.1.7, B.1.351, and P.1 lineages, this highly positively charged residue (K417) is changed by chargeless residues (Asp or Thr) (**Table 2**), which abolish the cation–pi interaction, yielding low HLA-binding affinity. Similarly, in the NYN_S/HLA-A*24:02 complex, L452 mediating hydrophobic interactions is primarily responsible for the high-affinity binding and stabilization of this complex (**Figure 2B**), despite the presence of salt bridge interactions (99–101). The non-silence mutation of leucine to arginine in VOCs abolishes the hydrophobic interactions, resulting in a loss of affinity for HLA. Overall, mutated peptides cannot be loaded onto their respective HLA molecules and presented by MHC-I to CD8^+^ T-cells, resulting in the inactivation of cytotoxic responses (CD8^+^ CTLs).

#### 5.2.2 Mutations disrupt epitope–HLA complex recognition by TCRs

Wu et al. (27) solved two CD8^+^ T-cell epitope structures in complex with HLA-A2 (RLQ–HLA-A*02:01 and YLQ–HLA-A*02:01) and with their respective TCRs (RLQ3–RLQ–HLA-A*02:01 and YLQ7–YLQ–HLA-A*02:01). As discussed previously, the wild-type RLQ and YLQ peptides form strong and stable complexes, mainly stabilized by Leu-1001 and Thr-274, respectively (**Figures 2C, D**). Similarly, RLQ3 and YLQ7 TCRs form strong and stable complexes with RLQ–HLA-A*02:01 and YLQ–HLA-A*02:01, respectively, featured by Arg-1000, Ser-1003, Leu-1004, Gln-1005, Thr-1006, and Tyr-1007 for the RLQ3–RLQ–HLA-A*02:01 complex and Tyr-269, Pro-272, Arg-273, Thr-274, Phe-275, and Leu-277 for the YLQ7–YLQ–HLA-A*02:01 complex (**Figures 2C, D**, **3**), which mediate binding with TCRs. These structural characteristics of the HLA–peptide–TCR complexes perfectly align with the respective ability of TCRs to interact with HLA peptides and activate CD8^+^ T-cell responses. Interestingly, TCR RLQ3 and YLQ7 could not recognize homologous RLQs and YLQs from other sarbecoviruses, nor could they recognize dominant SARS-CoV-2 RLQ and YLQ peptide variants and induce a CD8^+^ T-cell response. The most dominant variants in the SARS-CoV-2 VOCs include Q1005H and T1006I for RLQ, and L270F and P272L for YLQ (**Table 2**). Thus, it was evidenced that mutants T1006I and L270F (25) drastically reduce the binding affinity of RLQ and YLQ to and their loading onto HLA-A2s, as the stabilized interactions mediated by T1006 in RLQ–HLA-A2 and L270 in YLQ–HLA-A2 are abolished (**Figures 2C, D**, **3**).

In HLA–peptide–TCR complexes, mutation T1006I impairs HLA-A2–RLQ recognition by TCR RLQ3 because, together with Gln-1005, Thr-1006 are principal stabilizers of RLQ3–RLQ–HLA-A*02:01 complex as they establish the strongest bonds, including hydrogen and van der Waals interactions in the structure (27) (**Figures 3A, B**). Similarly, in the YLQ7–YLQ–HLA-A*02:01 structure, Arg-273 and Thr-274 form the most and strongest contacts (38/62 van der Waals and 14/15 contacts) with YLQ7 (**Figures 3B–E**); thus, mutations in one or both of these residues completely disrupt the recognition of YLQ–HLA-A*02:01 by YLQ7. Taken together, these mutations disrupt not only epitope binding to HLA-A2 but also and especially HLA-A2–epitope binding to TCRs, which corroborates the inability of mutated RLQ and YLQ to activate CD8^+^ T-cell responses.

This phenomenon of selective mutations at specific antigenic sites or at CD8^+^ T-cell epitopes aiming to reduce affinity to HLA molecules and TCRs, demonstrated for these four amino acids, is commonly used by all VOCs to hamper immune response activation and successfully escape from it (**Figure 4**; **Table 2**). **Table 2** presents the validated CD8^+^ T-cell epitopes for which mutations induce a reduced T-cell immunity against VOCs and virus immune escape. The direct impact of CD8^+^ T-cell inactivation through mutated CD8^+^ T-cell-dominant epitopes is the loss of chemotactic mechanisms, allowing the production and accumulation of proinflammatory cytokines and the recruitment of immune cells involved in eliminating the VOC-infected cells (25).

### 5.3 SARS-CoV-2 VOCs induce lymphopenia by targeting T-cells and lymphoid organs

Another mechanism suggested to be adopted by SARS-CoV-2 VOCs to escape the CD8^+^ T-cell response includes the direct destruction of the T-cells and/or the damage of the lymphoid organs producing T-cells. In fact, during infection, SARS-CoV-2 targets and infects the lymphocytes, which they kill (102), yielding lymphocyte depletion, known as lymphopenia (or lymphocytopenia), which is a common characteristic of COVID-19 severity (102, 103). More interestingly, lymphopenia is also well explained by the fact that SARS-CoV-2 may trigger the production of proinflammatory cytokines, including IL-1β, IL-8, IL-6, CXCL8/IL-8, TNF, and CXCL10/IP-10 in infected macrophages and dendritic cells, which directly decimate lymphoid organs, including spleen, lymph nodes, bone marrow, and thymus, and therefore blocking T-cell (including CD8^+^ T-cell) activation (61, 64, 65, 102–104). Specifically, postmortem autopsies from spleens of deceased COVID-19 patients showed that CD8^+^ T-cells were extremely low in all patients, and inflammatory cytokines (IL-6, IL-8, and IL-10) were increased, along with severe spleen tissue damage. Also, necrosis and lymphocyte apoptosis were detected in most patients, whereas artery thrombosis and spleen damage were observed in all patients (103, 104). This suggests that SARS-CoV-2 infection directly damaged the spleen and atrophied lymphoid follicles, yielding low production of CD8^+^ T-cells and NK cells.

Moreover, a positive link has been established between T-cell death (or exhaustion) and an increased expression of immune checkpoint inhibitor proteins (PD-1/PD-L1) at the CD4^+^ and CD8^+^ T-cell surface in severe SARS-CoV-2 patients (105, 106). For instance, in SARS-CoV-2 patients, it was demonstrated that overexpression of PD-1 and PD-L1 induces the activation of the PD-1/PD-L1 signaling pathway, which downregulates the activation of effector T-cell responses through a programmed T-cell death mechanism and predicts COVID-19 severity (52, 106). In the study of Ronchi et al. (106), severe COVID-19 patients and patients who died from COVID-19 had a depleted T-cell response, especially CD8^+^ T-cells, and a high viral load with a hyperexpression of PD-L1 by pneumocytes. This suggests that SARS-CoV-2 infection induces upregulation of a PD-1/PD-L1 signaling pathway, which is responsible for the T-cell death and CD8^+^ T-cell immune escape. Consequently, the virus gains the advantage of this state being more threatening. These mechanisms might be enhanced in SARS-CoV-2 VOC infection cases given the successful and noteworthy evasion by VOCs of CD8^+^ T-cell response. Future studies should address the contribution of SARS-CoV-2 VOCs to programmed lymphocyte death and lymphoid organ damage.

## 6 Discussion and concluding remarks

Humoral immunity to SARS-CoV-2 is widely studied as it plays an essential role in virus recognition and neutralization through neutralizing antibodies. However, this role is only limited to extracellular environmental scenarios. Moreover, memory antibodies and B-cells are relatively short-lived, non-persistent over the years, and become undetectable after 4 years post-infection (107, 108), compared with T-cells that can last longer and persist for more than a decade (66). Moreover, occasionally and paradoxically, antibodies can increase virus severity through the antibody-dependent enhancement (ADE) phenomenon. These limits would push scientists to focus on cellular immune response, which plays an important role, too, as it is involved in the destruction and eradication of the infected cells carrying virus particles. Therefore, cellular immunity is as essential as humoral immunity in infection clearance.

Despite the efforts put toward the development of strategies to fight against SARS-CoV-2 infections and COVID-19, we still have a long way to go because of the emergence of new SARS-CoV-2 variants, including VOCs, which are more virulent and severe than authentic SARS-CoV-2, and especially resistant to CPs, SARS-CoV-2-specific NmAbs, and the current vaccines (13, 14). Remarkably, since the beginning of the outbreak, the pattern of the COVID-19 pandemic shows surges in new cases and fatalities, followed by declines, and as of now, the world faces a new COVID-19 wave since January 2022 that peaked early in February (https://covid19.who.int/; https://www.worldometers.info/coronavirus/). Interestingly, most of these new cases are caused by the new SARS-CoV-2 strains (1–3) classified by the WHO and CDC as VOCs (https://www.cdc.gov/coronavirus/2019-ncov/variants/variant-classifications.html#anchor_1632154493691). Indeed, it is the non-silent mutations occurring in SARS-CoV-2 that confer to VOCs the ability to escape from innate and adaptive immunity, especially from CD8^+^ T-cell immunity, and exert their virulence in humans (11, 12, 14) (**Table 1**).

While some studies have demonstrated highly conserved CD8^+^ and CD4^+^ T-cell epitopes in VOCs (71, 72), with evidence of T-cell response similarities between WT and mutants (63), it is important to note that the few mutational rates present in structural and non-structural gene products of VOCs, specifically within the CD8^+^ T-cell epitopes, can exert a cellular immune escape, leading to fatalities. Given the small size of CD8^+^ T-cell epitopes (8 to 10 amino acids), a single mutation within these epitopes is sufficient to disrupt CTL response (62, 85). This needs to be taken into account given the fact that people who present deficient or non-functional (or non-active) CD8^+^ T-cells, even with stable CD4^+^ T-cell response, are vulnerable and susceptible to COVID-19 severity (76–78, 80–83, 109). Two shreds of evidence have been presented here: (i) high CD4^+^ T-cell titers but low CD8^+^ T-cell titers were found in critically ill COVID-19 patients infected with VOCs, whereas the opposite was found in mild and recovered COVID-19 patients (53–55); (ii) SARS-CoV-2 recovered patients have genetically conserved T-cell immunity, which can also specifically recognize VOCs; however, these patients can still be reinfected by new SARS-CoV-2 VOCs and, more interestingly, they can experience severe acute respiratory distress syndrome (ARDS) (76, 80–83, 109). These suggest that mutations that occurred in WT SARS-CoV-2 leading to VOCs have negative effects on the production of CD8^+^ T-cells, and VOCs can still escape from SARS-CoV-2–specific preexisting cell immunity (80, 81), which, even at high titers, may not be as effective as it would be if reinfection occurred with the original WT SARS-CoV-2.

Among the evasion pathways that SARS-CoV-2 VOCs may adopt to escape from natural and/or vaccine-induced CD8^+^ T-cell immune responses specific to WT SARS-CoV-2, we summarize three possible mechanisms:

• SARS-CoV-2 VOCs also adopt and enhance the SARS-CoV-2 mechanism of activation of the autophagy pathway and the lysosomal degradation of MHC-I, which highly decreases the activation of the CD8^+^ CTL response due to mutations of the ORF8 (**Figure 1**) (86, 96);
• Mutations in CD8^+^ T-cell epitopes specific to SARS-CoV-2 proteins induce a loss of affinity and cannot be loaded onto HLA-A molecules, which results in a lack of TCR recognition and cytotoxicity activation (**Figures 2** **–** **4**) (27, 80, 81, 87);
• SARS-CoV-2 VOCs may induce enhanced direct destruction of the T-cells and/or damage of the lymphoid organs producing T-cells, specifically CD8^+^ T-cells, through the hyperactivation of the PD-1/PD-L1 signaling pathway.

While mutations in SARS-CoV-2 SP and NSP, specifically in CD8^+^ T-cell epitopes, have been demonstrated to induce VOC immune escape through the inactivation or downregulation of CTL, future studies should address the contribution of SARS-CoV-2 VOCs, especially mutations in CD8^+^ T-cell epitopes, on programmed lymphocyte death and lymphoid organ damage, specifically in the overexpression of PD-1 and PD-L1 on CD8^+^ T-cells.

From a reverse point of view, considering studies claiming that reported mutations occurring in CD8^+^ T epitopes have no effects on WT SARS-CoV-2 T-cell response (71, 72, 80, 81), specifically on CD8^+^ T-cell activation and are barely preserved within VOCs, this could hypothetically imply that these mutations may create new specific VOC CD8^+^ T-cell epitopes (25, 74, 75, 85), which might contribute to an effective but delayed clearance of VOCs. For instance, recovered patients from WT SARS-CoV-2 infection or WT SARS-CoV-2 vaccinees or both acquired a protective memory T-cell immunity fully against WT SARS-CoV-2 [with a negligible reinfection absolute rate of 0%–1.1% (68)] but more than 50% reduced against new variants (80, 81, 109). In the case of new infection with VOCs, this less than 50% T-cell immunity, especially CD8^+^ T-cells (80, 81, 109), may not be strong enough to eliminate the new variants in reasonable kinetics as the variant may also escape from preexisting immunity. Thus, hypothetical new CD8^+^ T-cell epitopes would be loaded onto corresponding HLA-I molecules and trigger new and specific T-cell activation for a complete—delayed—VOC clearance. Studies by Qiu et al. (75) and Elisa Guo and Hailong Guo (74) demonstrated the possibility of new CD8^+^ T-cell epitopes from mutated epitopes of SARS-CoV-2, with the ability to increase T-cell activation marker CD69 and CD137 and induce low titer CD8^+^ T-cell response specific to the mutants, but then, no more specific to the WT SARS-CoV-2. Future studies need to assess the possibility of new epitopes from SARS-CoV-2 VOC infections and their effectiveness in the clearance of SARS-CoV-2 VOCs. These studies would help in developing variant-specific vaccines.

Additionally, other studies have raised the conclusion that despite mutations occurring in SARS-CoV-2, which are responsible for SARS-CoV-2 emerging variants (including VOCs), recovered WT SARS-CoV-2 individuals and WT SARS-CoV-2–specific vaccinees retain immunity that cross-reacts with new variants and may clear the VOC infections and prevent them from severe forms of COVID-19 (68, 73). However, this—early—immunity effectiveness might be mainly attributed to memory CD4^+^ T-cells and, to a lesser extent, to memory B-cells and antibodies, but probably not to memory CD8^+^ T-cells. This is because, as described in **Section 4**, mutated epitopes carried by VOCs may no longer be recognized by preexisting CD8^+^ T-cell immunity, as mutations in SARS-CoV-2 negatively affect mainly CD8^+^ T-cell epitopes that are more vulnerable (62, 75), but not CD4^+^ T-cell epitopes for which the same preexisting SARS-CoV-2 immune response still retains efficacy against mutants and may appropriately reduce VOC infection-associated severity (72). Consequently, we suggested that the clearance of VOC infections later on without intensive care admission could be possibly attributed to the development of new CD8^+^ T-cell epitopes specific to variants, together with the conserved preexisting CD4^+^ T-cell, which aligns with the global pattern of the COVID-19 pandemic [surges in new cases followed by prevalence declines months later (https://covid19.who.int/)].

In conclusion, to block the CTL-mediated cellular immune escape by SARS-CoV-2 VOCs, studies should focus on the development of new vaccines (such as RNA vaccines, which are known to promote the activation of cellular immunity) and especially on how to boost the CD8^+^ T-cell response against VOCs (110, 111). Besides RNA vaccines, a better alternative for next-generation vaccines includes epitopes-based vaccines. By focusing on non-structural proteins and spike and nucleocapsid protein domains of SARS-CoV-2 that are relatively less mutated or highly conserved, numerous studies demonstrated dominant CD8^+^ CTL epitopes specific for HLA-A*24:02 and HLA-A*02:01 genotypes, with a relatively low or zero divergence rate, that can be targeted for developing wild spectrum COVID-19 vaccines, effective against any SARS-CoV-2 variants—and extensively against sarbecoviruses—with the ability to induce neutralizing antibodies and activate specific CD8^+^ CTLs (27, 92, 112–115). For example, considering five randomly evidenced CD8^+^ CTL-specific epitopes with low/no mutational rates, such as FLNGSCGSV and VLAWLYAAV (91), PDPSKPSKR, DPSKPSKRS, and QTQTNSPRR (113), new-generation epitope-based vaccines might consist of developing a multivalent-epitope–based cocktail against SARS-CoV-2 from these five epitopes, with peptide carriers and/or intramolecular adjuvants. Besides boosting CD8^+^ T-cell response, one of the most attractive advantages of such multiple epitope-based vaccines includes the ability to reduce the potential of new SARS-CoV-2 emerging variant development. More interestingly, these epitope-based vaccines have more benefits, including time- and cost-effectiveness, maximal therapeutic efficacy (enhanced antigenicity and immunogenicity), and well-tolerability with minimal adverse effects (113, 115, 116).

Also, reports have demonstrated that a knockdown or a complete deletion of ORF8 activates surface MHC-I proper expression and significantly reduces immune escape (82, 96), suggesting that inhibiting ORF8 of SARS-CoV-2 constitutes a way to enhance CD8^+^ T-cell efficacy against SARS-CoV-2 VOC infections.

## Author contributions

AK conceived the presented idea, extracted the data, wrote the original draft, and formatted the manuscript for submission. FB and ZN reviewed and edited the final version for publication. TJ conceptualized the main idea, provided resources and financial assistance during the whole study, and supervised the whole paper. All authors contributed to the article and approved the submitted version.

## Funding

TJ is supported by the Strategic Priority Research Program of the Chinese Academy of Sciences (Grant No. XDB29030104), the National Natural Science Fund (Grant No.: 31870731), the Fundamental Research Funds for the Central Universities, and the 100 Talents Programme of The Chinese Academy of Sciences. AK is supported by the Chinese National Postdoctorate Subvention. ZNN is supported by a Chinese government scholarship.

## Acknowledgments

We apologize in advance to colleagues whose work was overlooked because of length limitations or by our own ignorance.

## Conflict of interest

The authors declare that the research was conducted in the absence of any commercial or financial relationships that could be construed as a potential conflict of interest.

## Publisher’s note

All claims expressed in this article are solely those of the authors and do not necessarily represent those of their affiliated organizations, or those of the publisher, the editors and the reviewers. Any product that may be evaluated in this article, or claim that may be made by its manufacturer, is not guaranteed or endorsed by the publisher.
